# Supplementary material for: Controlling the thermoelectric effect by mechanical manipulation of the electron’s quantum phase in atomic junctions
Source: Sci Rep. 2017 Aug 11;7:7949. doi: 10.1038/s41598-017-08553-2 (PMC5554135; doi:10.1038/s41598-017-08553-2)
Supplement: Supplementary file 1 — Supplementary information [file 41598_2017_8553_MOESM1_ESM.pdf]

Supplementary Information

Controlling the thermoelectric effect by mechanical manipulation of the electron's quantum phase in atomic junctions

Akira Aiba[1], Firuz Demir[2,3], Satoshi Kaneko[1], Shintaro Fujii[1], Tomoaki Nishino[1], Kazuhito Tsukagoshi[4], Alireza Saffarzadeh[2,5], George Kirczenow[2]*, Manabu Kiguchi[1]*

[1]Department of Chemistry, Graduate School of Science and Engineering, Tokyo Institute of Technology, Ookayama, Meguro-ku, Tokyo 152-8551, Japan

[2]Department of Physics, Simon Fraser University, Burnaby, British Columbia, Canada V5A 1S6

[3]Physics Department, Khalifa University of Science and Technology, P.O. Box 127788, Abu Dhabi, UAE

[4]National Institute for Materials Science, Tsukuba, Ibaraki 305-0044, Japan

[5]Department of Physics, Payame Noor University, P.O. Box 19395-3697 Tehran, Iran

*Correspondence and requests for materials should be addressed to G.K.(E-mail: kirczeno@sfu.ca) and M.K. (kiguti@chem.titech.ac.jp)

Table of Contents

S1. **Conductance and thermoelectric voltage measurement.**

S2. **Evaluation of the thermopower of the Au atomic junction.**

S3. **Thermoelectric properties of the Au atomic junction.**

S4. **Example of conductance and thermoelectric voltage switch.**

S5. **Origin of the change in the thermoelectric voltage due to mechanical strain.**

S1. Conductance and thermoelectric voltage measurement

The measurements were performed using the mechanically controllable break junction (MCBJ) technique. Figure S1a shows the MCBJ sample measured in the present study. Two Pt resistive heaters (width 200 μm, length 8 mm, thickness 100–150 nm) were deposited on polyimide tape via magnetron sputtering. The tops of the heaters were covered with polyimide tape for electrical insulation. The covered Pt heaters and the phosphor bronze substrate were thermally insulated with fluoroplastic saturated glass cloth tape. A notched Au wire (0.1 mm diameter, 99.99% purity) was fixed with epoxy adhesive (Stycast 2850FT) on the covered Pt heaters. The RuO_2 thermometers were fixed on the Au wire at both ends, as shown in Fig. S1. The MCBJ sample was mounted in a regular MCBJ setup inside a cryogenic vacuum chamber. The conductance and thermoelectric voltage were measured in four steps at low temperatures (10~50 K), as follows: 1) The voltage was measured at zero current bias. 2) The current was measured at a voltage of +50 mV. 3) The voltage was measured at zero current bias. 4) The current was measured at a voltage of -50 mV. Each cycle took approximately 0.2 s. The current and voltage were measured with a source measure unit (Keithley 2612A). The conductance was obtained from the current difference for the two voltage polarities, and the thermoelectric voltage was obtained from the average of two voltage values at zero bias current. Figure S1b shows the conductance change of the Au contact during stretching of the contact, and Fig. S1c shows the corresponding conductance histogram. The last step at 1 G_0 ($2e^2/h$) in the conductance trace and the 1 G_0 peak in the conductance histogram correspond to the formation of the Au atomic junction. There is a temperature difference between the junction and each side of the contact, where the thermometer is attached, caused by the heat flow through the glass tape, conducting wires attached to the

sample, and conducting wires attached to the thermometer, heat flow between the stem part of the electrode and atomic junction, and heat dissipation in the atomic junction[1,2]. The estimation of these heat flows and heat dissipation is very difficult, and thus, it is hard to evaluate the actual temperature difference over the junction. A previously reported study determined the actual temperature difference over the contact to be a fraction, 0.3~0.5, of the measured temperature difference using the bulk thermopower of pure gold or scanning thermal microscopy[3-5]. The present experimental setup is the same as in this previously study. So, the actual temperature difference over the contact can be obtained by multiplying the experimentally obtained temperature difference by a factor of 0.3~0.5.

We calibrated the displacement by the following process. In the MCBJ, the setup itself acts as a reduction gear for the motion of the piezo element (δx) with respect to the relative displacement of the two electrodes (δy). For the ideal case of homogeneous strain in the bending beam, the displacement ratio (r) between δy and δx is given by $r = \frac{\delta y}{\delta x} = \frac{6tu}{l^2}$, where t, u and l are the thickness of the bending beam, the distance between the epoxy droplets and the distance between the two counter supports, respectively. The displacement ratio was 2×10^{-3} for the present setup, with l=20mm, t=1mm, u=0.1 mm. We also calibrated the displacement ratio based on the length histogram of the clean Au atomic chain. A sequence of peaks was observed in the length histogram. The interval of the peaks was defined as 0.255 nm, which corresponds to the Au-Au distance of a clean Au atomic wire.

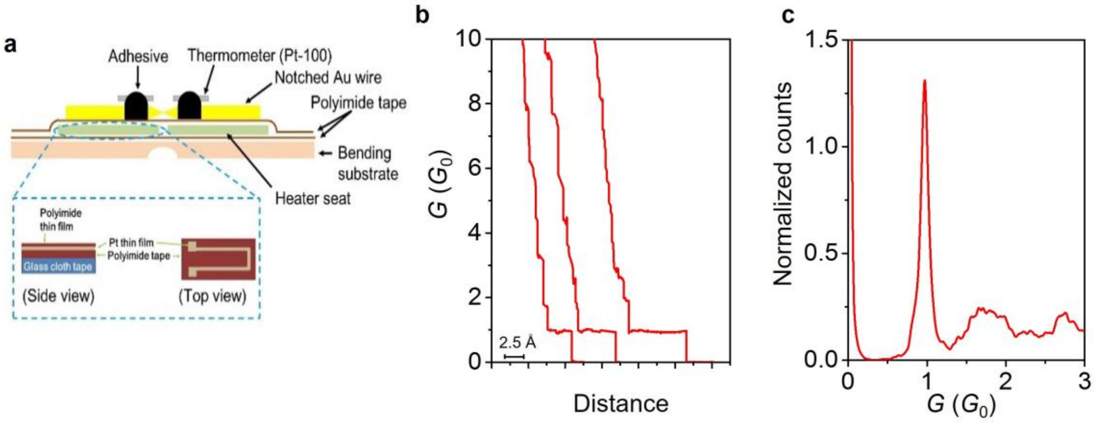

Fig. S1. Experimental setup for simultaneous measurement of the electrical conductance and thermoelectric voltage of the Au atomic junction. (a) Schematic diagram of the modified MCBJ setup used for simultaneous measurement of conductance and thermoelectric voltage. (b) Conductance change of the Au atomic junction with stretching (c) Conductance histogram of the Au atomic junction.

S2. Evaluation of the thermopower of the Au atomic junction

Figure S2 shows a schematic of the configuration of the circuit. Points at 0, 1, 2, 3 correspond to the anchor pad, low temperature side of the junction, high temperature side of the junction, and anchor pad, respectively. Both anchor pads are attached to the liquid He insert, and thus the temperatures of the anchor pads are the same in this circuit. The thermopower is defined as

$$-\Delta V = S\Delta T \quad (1)$$

On integration Eq. 1, the thermoelectric voltage is given by

$$V_1 - V_0 = -S_{bulk-Au}(T_1 - T_0) \quad (2)$$

$$V_2 - V_1 = -S_{Junction}(T_2 - T_1) \quad (3)$$

$$V_3 - V_2 = -S_{bulk-Au}(T_0 - T_2) \quad (4)$$

Where $S_{Junction}$ and $S_{bulk\text{-}Au}$ are the thermopower of the Au atomic junction and bulk Au, respectively. Upon adding Eq. 2-4:

$V_3 - V_0 = (S_{bulk-Au} - S_{Junction})(T_2 - T_1)$ (5)

From which the junction thermopower is obtained by

$$S_{Junction} = S_{bulk-Au} - \frac{V_3 - V_0}{T_2 - T_1} \qquad (6)$$

The thermopower of bulk Au is known to be

$S_{bulk-Au}(T) = 4.76 \times 10^{-2} T - 0.518\ (\mu V K^{-1})$ (7)

for the temperature regime of 20 K to 40 K [4,5]. The thermopower of the Au atomic junction is obtained in this study based on Eq(6) and (7).

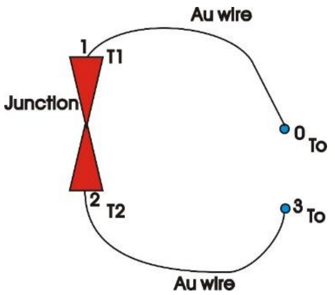

Fig. S2. Schematic showing the configuration of the circuit.

S3. Thermoelectric properties of the Au atomic junction

The thermoelectric voltage of the Au atomic junction fluctuated with the sample, as shown in Fig. 1c. In the case of Fig. 1b, one of the examples of the conductance and thermoelectric voltage traces, the thermoelectric voltage was negative around 1 G_0, corresponding to the mono atomic contact. For another sample, the thermoelectric voltage was positive around 1 G_0. In order to grasp the general trend, the statistical analysis was

performed for the thermoelectric voltage of the Au atomic junction. Figure S3a shows the average thermoelectric voltage of the Au mono atomic contact as a function of the temperature difference across the junction (ΔT). The thermoelectric voltage of the Au atomic junction (V_T) is represented by

$$V_T = (S_{Junction} - S_{bulk-Au})\Delta T \quad (8)$$

where $S_{Junction}$ and $S_{\text{bulk-Au}}$ are the thermopower of the Au atomic junction and bulk Au. The $S_{\text{bulk-Au}}$ is 0.7 μV/K around 25 K[4,5]. Within the small temperature difference regime (ΔT <10 K), the thermopower is regarded as having a constant value; the thermoelectric voltage increases with the temperature difference. From the slope, the thermopower of the Au atomic junction is evaluated to be -0.7 μV/K, whose absolute value is much smaller than that of a single molecular junction and close to zero[6]. The standard deviation of thermoelectric voltage increases with the temperature difference (Fig. S3b).

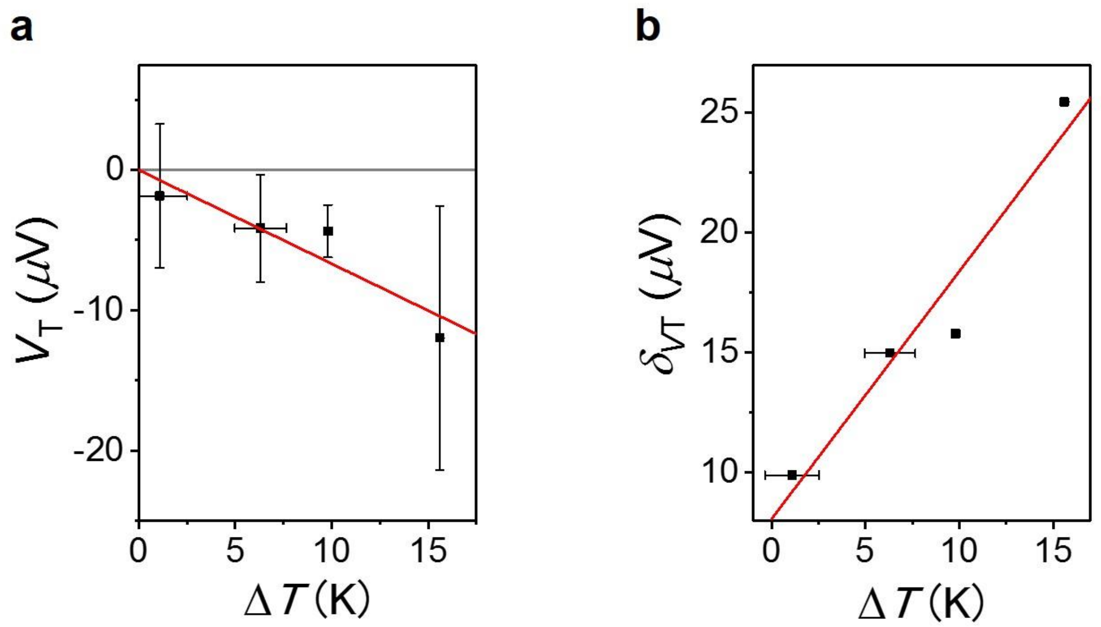

Fig. S3. Thermoelectric voltage of the Au atomic junction. (a) The average (V_T) and (b) standard deviation (δ_{VT}) of the thermoelectric voltage of the Au atomic junction as a

function of the temperature difference between the junction (ΔT). The average sample temperature was 24 K. The ΔT-V_T and ΔT-δ_{VT} plots were obtained from 30000 data points for three individual samples.

S4. Example of conductance and thermoelectric voltage switch

Figure S4 shows examples of thermoelectric switching of the Au atomic junction. Figure S4c and d present results of mono Au atomic junctions whose conductances are below 1 G_0. The power factor of the Au atomic junction is also shown in the figure. The power factor (P) of the Au atomic junction is obtained from Eq. (9):

$$P_{junction} = S_{junction}{}^2 G \quad (9)$$

where $S_{Junction}$ and G are the thermopower and the conductance of the Au atomic junction [7]. The thermoelectric voltage and conductance switch reversibly between two distinct states. The reproducibility of the conductance and thermoelectric voltage switching behavior improves with the elongation/compression distance in the regime of 0.1~0.7 nm. The modulations of the conductance and thermoelectric voltage are subject to change as the process is repeated (Decrease: Fig. S4b, Constant: Fig. S4a, Increase: Fig. S4c). Although the slight mechanical modulation and low temperature realize the reversible structural change of the Au atomic junction within limited cycles, the structure of the Au atomic junction and distribution of defects around the junction gradually change as the processes is repeated, which causes the observed change in the amplitude of the modulation.

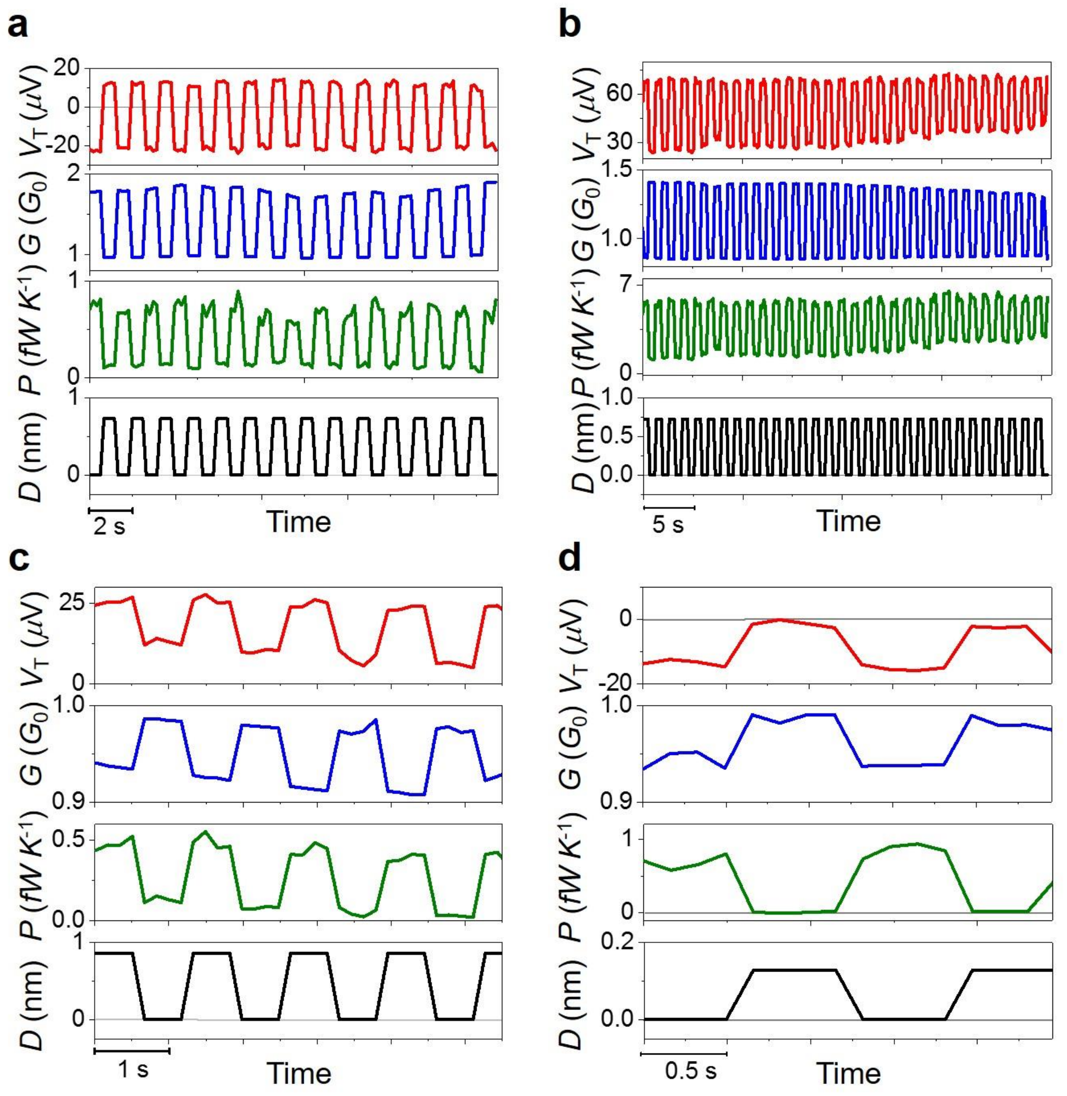

Fig. S4. Thermoelectric switching of the Au atomic junction. (a) T_{ave}: 25 K, ΔT:9.6 K (b) T_{ave}:23 K, ΔT =7.4 K, (c) T_{ave} : 25 K, ΔT = 10 K, (d) T_{ave}: 25 K, ΔT =4.4 K.

S5. Origin of the change in the thermoelectric voltage due to mechanical strain

Figure 3(c-h) show the calculated model structures, transmission curves and $\Delta V/\Delta T$ for model clusters composed of 240 Au atoms. In order to display graphically the dependence of the phase of a typical transport state Ψ^{α} on the position in the junction within a tight-

binding model, it is useful to consider the value Ψ^{α} (rather than $|\Psi^{\alpha}|^2$) averaged over each atomic site, since this average contains the information about the variation of phase of the wave from site to site. Our transport calculations include the 6s, 6p and 5d valence orbitals of Au atoms in the basis. Thus on each site Ψ^{α} is a linear combination of 6s, 6p and 5d atomic orbitals. However, each atomic orbital is of the form $R_{nl}(r)Y_{lm}(\theta,\phi)$, and the angular average of the spherical harmonics $Y_{lm}(\theta,\phi)$ is not zero only for s orbitals. Therefore the 6p and 5d atomic orbitals do not contribute to the site average of Ψ^{α}. For this reason in order to display the phase of the wave function we consider $<6s|\Psi^{\alpha}>$, the projection of the state on the 6s valence orbitals of the atoms of interest. This also makes physical sense because the 6s atomic orbital is the closest in energy to the Fermi level of gold.

The shift to lower energies of the α and β resonances when the structure changes from that in Fig.3c to that in Fig.3d can now be understood as follows: In Fig.3c and d we show as insets $\mathrm{Re}\left(\left\langle 6s|\Psi^{\alpha}\right\rangle\right)$, the real part of a typical transport state $\left|\Psi^{\alpha}\right\rangle$ (a solution of the Lippman-Schwinger equation 2 at the Fermi energy) projected onto the 6s valence orbitals of the Au atoms of the junction. The purple dots show the values of $\mathrm{Re}\left(\left\langle 6s|\Psi^{\alpha}\right\rangle\right)$ on the two central Au atoms and the averages of $\mathrm{Re}\left(\left\langle 6s|\Psi^{\alpha}\right\rangle\right)$ over the three Au atoms at either end of the junction. The purple curves are guides to the eye and the green lines indicate $\mathrm{Re}\left(\left\langle 6s|\Psi^{\alpha}\right\rangle\right)=0$. These plots indicate that the phase φ of the electron wave function varies in a regular way as the junction is traversed.

The phase differences $\Delta\varphi$ developed across the junction by the wave functions are expected to influence the electronic quantum interference patterns throughout both electrodes as well as in the junction and therefore to influence the energies at which the transmission resonances in Fig.3e and f occur. An appropriate acoustic analogy is that adding to the length of an organ pipe or guitar string lowers the resonant frequencies of the instrument.

By setting $\rho e^{i\phi} = \left\langle 6s \middle| \Psi^{\alpha} \right\rangle$ for real ρ and ϕ, we estimate the phase differences $\Delta\varphi$ developed across the junction by the wave functions shown in the insets of Fig.3c and d to be 2.23π, and 2.36π respectively. Thus $\Delta\varphi$ is higher while the energies of the resonances α and β are lower for the structure in Fig.3d than for the structure in Fig.3c.

We can account qualitatively for the above finding that the resonance energies are lower for higher $\Delta\varphi$ if we note that gold, at the simplest level of modeling, can be regarded as a metal with a positive effective mass at the Fermi energy. For such a material the electron de Broglie wavelength is larger at lower energies. A resonant electronic state is characterized by the phase differences of its wave function between locations in the two electrodes. If, for a particular resonant state, the junction between the two electrodes changes in such a way that the electron phase difference $\Delta\varphi$ across the junction increases, then for the system to remain on resonance, phase differences between the junction's boundaries and the locations of scatterers in each electrode should decrease to compensate for the increase in $\Delta\varphi$ across the junction. This can occur if the resonance shifts to a lower energy where the electron de Broglie wavelength in the electrodes is larger and hence phase differences between different points within the electrodes are smaller. Thus an increase in $\Delta\varphi$ across the junction may be expected to result in a shift of transmission resonances to lower energies and vice versa.

References

1 Tsutsui, M., Kurokawa, S. & Sakai, A. Bias-induced local heating in atom-sized metal contacts at 77 K. *Appl. Phys. Lett.* **90**, 133121, (2007).

2 Lee, W. *et al.* Heat dissipation in atomic-scale junctions. *Nature* **498**, 209, (2013).

3 Kim, Y., Jeong, W., Kim, K., Lee, W. & Reddy, P. Electrostatic control of thermoelectricity in molecular junctions. *Nat. Nanotechnol.* **9**, 881-885, (2014).

4 Pearson, W. B. Survey of Thermoelectric studies of the group-I metals at low temperatures carried out at the National-Research-Laboratories, Ottawa. *Sov. Phys. - Sol. State* **3**, 1024-1033, (1961).

5 Guenault, A. M. & Hawksworth, D. G. Thermoelectric-power of pure noble-metals at low-temperatures. *J. Phys. F. Met. Phys.* **7**, L219-L222, (1977).

6 Evangeli, C. *et al.* Quantum thermopower of metallic atomic-size contacts at room temperature. *Nano Lett.* **15**, 1006-1011, (2015).

7 Rincon-Garcia, L. *et al.* Molecular design and control of fullerene-based bi-thermoelectric materials. *Nat. Mater.* **15**, 289-293, (2016).
